# Inhibition of primordial germ cell proliferation by the medaka male determining gene Dmrt1bY

**DOI:** 10.1186/1471-213X-7-99

**Published:** 2007-08-30

**Authors:** Amaury Herpin, Detlev Schindler, Anita Kraiss, Ute Hornung, Christoph Winkler, Manfred Schartl

**Affiliations:** 1 University of Wurzburg, Physiological Chemistry I, Biozentrum, Am Hubland, D-97074 Wurzburg, Germany; 2 University of Wurzburg, Department of Human Genetics, Biozentrum, Am Hubland, D-97074 Wurzburg, Germany; 3 University of Wurzburg, Rudolf-Virchow-Center for Experimental Biomedicine (DFG research Center), Versbacher Str. 9, D-97078 Wurzburg, Germany

## Abstract

**Background:**

*Dmrt1 *is a highly conserved gene involved in the determination and early differentiation phase of the primordial gonad in vertebrates. In the fish medaka *dmrt1bY*, a functional duplicate of the autosomal *dmrt1a *gene on the Y-chromosome, has been shown to be the master regulator of male gonadal development, comparable to *Sry *in mammals. In males mRNA and protein expression was observed before morphological sex differentiation in the somatic cells surrounding primordial germ cells (PGCs) of the gonadal anlage and later on exclusively in Sertoli cells. This suggested a role for *dmrt1bY *during male gonad and germ cell development.

**Results:**

We provide functional evidence that expression of *dmrt1bY *leads to negative regulation of PGC proliferation. Flow cytometric measurements revealed a G2 arrest of *dmrt1bY *expressing cells. Interestingly, also non-transfected cells displayed a significantly lower fraction of proliferating cells, pointing to a possible non-cell autonomous action of dmrt1bY. Injection of antisense morpholinos led to an increase of PGCs in genetically male embryos due to loss of proliferation inhibition.

**Conclusion:**

In medaka, *dmrt1bY *mediates a mitotic arrest of PGCs in males prior to testes differentiation at the sex determination stage. This occurs possibly *via *a cross-talk of Sertoli cells and PGCs.

## Background

Genetic evidence has suggested that the *dmrt1 *gene is an important regulator of male development in vertebrates. In humans, haploinsufficiency of the genomic region that includes *DMRT1 *and its paralogs *DMRT2 *and *DMRT3 *leads to XY male to female sex reversal [1]. In chicken and other avian species *Dmrt1 *is located on the Z chromosome, but absent from W, making it an excellent candidate for the male sex-determining gene of birds [2,3]. In the fish medaka (*Oryzias latipes*), which has XY-XX sex determination, a duplicated copy of *dmrt1*, designated *dmrt1bY *or *DMY*, on the Y-chromosome was shown to be the master regulator of male development [4,5], similar to *Sry *in mammals.

While all other known genes of the sex determination/sex differentiation cascade from mammals appear to be conserved only in vertebrates, and *Sry *even only in the placental mammals and marsupials, *dmrt1 *is exceptional, because it is a true homologue of the Drosophila *doublesex *(*dsx*) and *Caenorhabditis elegans *male abnormal *(mab-3) *genes. Both *dsx *and *mab-3 *are components of the genetic cascade that regulates sexual development. They encode transcription factors that control the expression of genes involved in the realization of the male or female phenotype. On the protein level they share a novel DNA binding motif, the doublesex and mab-3 homology (DM) domain that has been characterized as an intertwined Zn-finger [6]. The DM domain is also predicted for the proteins encoded by the vertebrate *dmrt *genes.

Both the *mab-3 *and *dsx *encoded proteins bind to similar DNA regulatory sequences of the control region of yolk protein genes and control their transcription [7,8]. Both regulate also the differentiation of sex-specific sense organs [9,10]. *Dsx *controls the sex-specific expression of *Fibroblast Growth Factor*, the *dachshund *and the *bric-a-brac *genes, thereby regulating development of genital structures and male-specific pigmentation. Furthermore, *dsx *modulates the response of cells of the genital disc to wingless and decapentaplegic signalling and in this respect regulates sex-specific growth and cell migration (for review see [11]).

Contrary to the situation in invertebrates, where much progress in the analysis of the biochemical function of *doublesex *and *mab-3 *as well as the cellular developmental biological effects has been made, such detailed information is still lacking for the vertebrate homologue. So far it is only known that in fish, frogs, turtles, alligators and chicken *dmrt1 *shows sexually dimorphic expression: it is expressed at higher levels in gonads that develop towards testes prior to sexual differentiation and continues to be active in adult male gonads, while it is downregulated in ovaries [12-18]. In mice *Dmrt1 *expression in the gonadal primordium is gradually lost in females, but is strongly upregulated during testes development and remains high in the adult organ [13]. Mutant male mice that lack *Dmrt1 *have multiple defects in postnatal testes development and are sterile [19]. On the cellular level the specific expression in Sertoli cells has attributed to *Dmrt1 *a function for these cells in the adult testes, while the expression in male germ cells in some studies remained enigmatic.

We have used the medaka to study the biochemical and cell biological function of Dmrt1 proteins. This small aquarium fish is comparable in its advantageous features for gene function analysis to the zebrafish. In addition, the duplicated version of *dmrt1*, the *dmrt1bY *gene, is the male determining gene in this species, being the only known master sex regulator besides *Sry *so far in vertebrates. *Dmrt1bY *is expressed in the pre-Sertoli cells of the developing testes [18,20], consistent with its predicted function to determine development of the testes from the undifferentiated gonad primordium. In adult males, the gene is co-expressed with its autosomal progenitor *dmrt1 *in Sertoli cells [4,12] which itself is not expressed at the time of gonad differentiation. Beside these differences in expression [18] there are also some differences in structure [20]. Although displaying a high identity at the amino acid level (more than 85%), phylogenetic analyzes have suggested that single amino acid changes could be largely responsible for the establishment of *dmrt1bY *as the male sex-determination gene in Medaka [20].

Interestingly, at the time of expression of *dmrt1bY *in the male gonad proimordium shortly before hatching, at stage 36, in both sexes PGCs migrate to the gonadal primordium where they actively proliferate in female but not in males [18,21]. As the number of PGCs at hatching roughly correlated with the inferred level of *dmrt1bY *expression the hypothesis was put forward that *dmrt1bY *may regulate PGC number [22]. The mechanisms how *dmrt1bY *might exert this function in unknown. Also, the the fact that the transcription factor Dmrt1bY, expressed in Sertoli cells, could act on PGCs has not been addressed. Here we show, using overexpression and downregulation by antisense morpholinos that *dmrt1bY *is a negative regulator of cell proliferation that is involved in the male specific cessation of primordial germ cell proliferation prior to testes differentiation in medaka.

## Results

### Dmrt1bY modifies cell cycle progression

**Figure 1:**
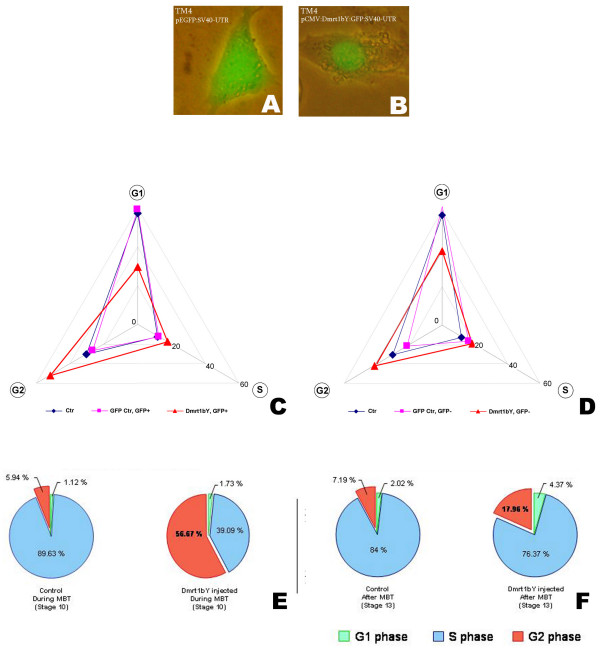
**Dmrt1bY overexpression modifies cell cycle pattern in cell culture as well as in live embryos**. A and B: GFP fluorescence is cytoplasmic in mouse TM4 cells transiently transfected with p CMV:EGFP-SV40UTR control plasmid (A) while nuclear localized when transfected with pCMV:Dmrt1bY:GFP-SV40UTR construct (B). C and D: Radar histograms representing the DNA content distribution of Dmrt1bY:GFP relative to GFP (control) transfected cells expressed in percentage. "Ctr" and "GFP Ctr, GFP+" represent GFP negative and positive control cells in the plate tranfected with control GFP plasmid; similarly "Dmrt1bY, GFP+" and "Dmrt1b, GFP-" represent GFP negative and positive cells In the plate tranfected with Dmrt1bY:GFP plasmid. E and F: Cell cycle distribution reflected by DNA content in control *versus *stage 10 (MBT) and post-MBT (stage 13) *Dmrt1bY*-injected embryos.

Considering that the first difference seen in medaka gonad development is a difference in PGC number due to propagation of PGCs in females but not in males we investigated whether Dmrt1bY plays a role in regulating cell proliferation and cell cycle progression. To analyze this, first mouse Sertoli TM4 [23] were transiently transfected with a Dmrt1bY:GFP expression construct under control of the CMV promoter (Fig. 1B). GFP transfected cells were used as controls (Fig. 1A). After 28–30 hours, TM4 cells transiently expressing the Dmrt1bY:GFP fusion protein (transfection efficiency 10 to 15%; Fig. 1C) exhibited a clear increase of cells in G2 phase (from 27 to 30% cells in G2 phase in the controls to 52% in Dmrt1bY expressing cells; Fig. 1C and additional figure 1A [see Additional file 1]. Raw data and statistical significance are shown in additional figure 1 [see Additional file 1]. While for both, controls (GFP positive and negative cells from the same plate) and *dmrt1bY*-transfected cells (GFP positive cells), the proportion of cells in S phase remained constant (around 12–17%), the increased number of cells in G2 phase was always balanced by a reduction of the proportion of cells in G1 phase (57–59% down to 30% respectively Fig. 1C and additional figures 1A and 1B) [see Additional file 1]. Interestingly, also the GFP negative (GFP-) cells from the cultures transfected with *Dmrt1bY*, showed reduced but similar G2 enrichment as the Dmrt1bY expressing GFP positive cells (Fig. 1D compared to 1C and additional figure 1A compared to additional figure 1B and 1D) [see Additional file 1]. Cells treated with serum conditioned by cells overexpressing Dmrt1bY did not display any change in cell cycle phase distribution (data not shown). Similar experiments in fish A2 cells revealed the same result [see additional file 1].

Taken together, these data suggest that Dmrt1bY affects cell proliferation by arresting cells in G2 phase through both a cell autonomous and a juxtacrine mechanism.

### Dmrt1bY inhibits cell propagation in early medaka embryos

**Figure 2:**
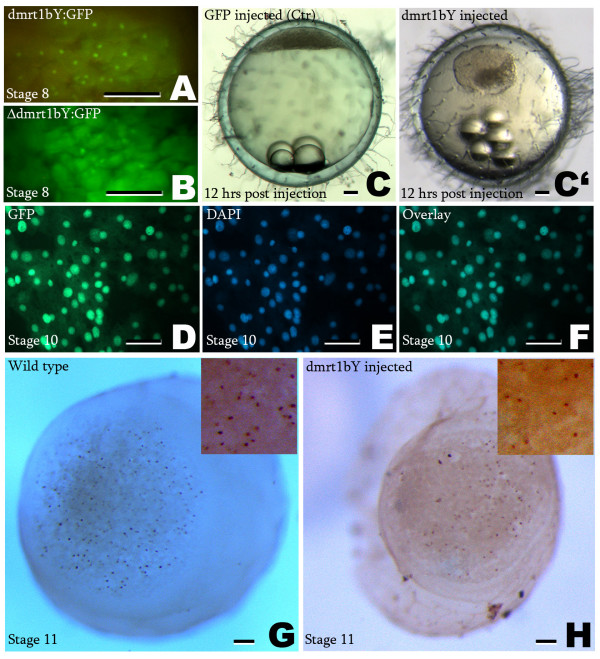
**Nuclear localization of Dmrt1bY:GFP and ΔDmrt1bY fusion proteins and modulation of cell proliferation by Dmrt1bY overexpression**. A and B: When capped RNAs encoding fusion protein of GFP with either Dmrt1bY (A) or ΔDmrt1bY (B) were injected into one cell stage embryos (75 ng/μL) strong nuclear localization is observed (A); mainly cytoplasmic (B) at stage 8. D, E and F: Green fluorescence produced by Dmrt1bY:GFP fusion protein colocalizes with DAPI staining of nuclei. GFP fluorescence (D), DAPI fluorescence (E), overlay (F). C and C': Embryos injected with high doses of Dmrt1bY:GFP, once arrested shortly after MBT, remain quiescent and become necrotic (C'). C: GFP-injected control embryo. G and H: Dmrt1bY injected embryos were stained for the mitotic marker phospho-Histone H3. Wild type embryo (G), Dmrt1bY injected embryo (H). Scale bars: 25 μM in A, B, D, E, F and 120 μM in C, C', G, H.

To confirm the inhibition of cell proliferation by Dmrt1bY observed in cell culture in an *in-vivo *situation capped mRNAs encoding fusion proteins of GFP with wild type Dmrt1bY (Dmrt1bY:GFP) or a version lacking the DNA binding DM domain (ΔDmrt1bY:GFP) were injected into embryos at the one cell stage (Fig. 2A and 2B). GFP RNA-injected control embryos showed exclusive cytoplasmic fluorescence (data not shown). Dmrt1bY-GFP fluorescence instead was observed preferentially in the nucleus (Fig. 2D,2E and 2F). This shows that the Dmrt1bY fusion protein was correctly translated and then properly imported into the nucleus. Surprisingly, although mutational analysis demonstrated the presence of a nuclear localization signal (NLS) in the DM domain of the zebrafish Dmrt2/Terra protein [24], our experiments show both cytoplasmic and nuclear accumulation of the ΔDmrt1bY:GFP fusion protein, which lacks the entire DM domain (Fig. 2B). This points to an additional nuclear targeting mechanism for Dmrt1bY outside of the DM domain.

To monitor for a possible proliferation effect, cell cleavages were followed from the 4 cell stage (stage 4) until shortly after mid-blastula transition (MBT), around stage 11. *Dmrt1bY:GFP *mRNA was injected at concentrations ranging from 5 ng/μL to 150 ng/μL. Doses below 10 ng/μL had no effect, while intermediate (between 20 and 75 ng/μL) and high doses (100 to 150 ng/μL) led to cessation of development at 90% epiboly/neurula stage and shortly after MBT (stage 10–11) respectively. Intermediate doses (20 to 75 ng/μL) induced developmental delay during early development around the MBT stage (stage 11). In embryos injected with high doses of *Dmrt1bY:GFP*, once arrested shortly after MBT (stage 11), blastula cells remained quiescent for 6–8 hours and then disintegrated (Fig. 2C and 2C'). For ΔDmrt1bY:GFP neither intermediate nor high doses had any effects on embryo cell proliferation indicating that the Dmrt1bY effect on early embryo cells is mediated by the putative DNA binding domain.

For cell cycle analysis, Medaka embryos were injected with either low doses (20 to 30 ng/μL of *Dmrt1bY:GFP *mRNA) or high doses (100 to 150 ng/μL of *Dmrt1bY:GFP *mRNA) and analyzed at stage 10 and 13 respectively. For both stages flow cytometry revealed a dramatic dose dependant increase of cells accumulated in G2 phase upon Dmrt1bY expression (Fig. 1E and 1F) that is balanced by a reduction of the proportion of cells in S phase. This *in vivo *result (Fig. 1E and 1F) corroborates the cell culture data (Fig. 1C and 1D).

### Dmrt1bY arrests cell proliferation modulates DNA replication and can lead to apoptosis

To examine whether the inhibitory effect of Dmrt1bY affects cell proliferation or causes cell death, one cell stage embryos injected with high dose (*Dmrt1bY:GFP*, 150 ng/μL) were allowed to develop until they stopped their development at stage 10–11 (mid blastula). At that stage apoptotic pathways usually become active [25]. Injected embryos were stained for the mitotic marker phospho-Histone H3 (pH3) (Fig. 2G and 2H) as well as by TUNEL assay for apoptosis [see additional figures 2A to 2F in additional file 2]. High Dmrt1bY:GFP doses led to an almost complete arrest of cell proliferation. Only 10 to 20% of the cells were positive for pH3, compared to controls, which displayed 70 to 80% proliferating cells (Fig. 2G and 2H). Embryos injected with intermediate doses of Dmrt1bY:GFP (50 ng/μL) that are able to pass through the MBT stage, were allowed to develop until late neurula (stage 18) and analysed the same way. Cell replication status was lower than in controls (35 to 45% vs. 70 to 80% pH3 positive cells). Analysis of apoptosis by TUNEL labelling revealed a statistically significant increase of apoptotic cells at the neurula stage (stage 18) [see additional figures 2C, 2D, 2E and 2F in additional file 2]. Most of these apoptotic cells were not in the embryo but were located at its periphery or spread all over the yolk sac [see additional figures 2E and 2F in additional file 2]. In summary, Dmrt1bY effects were clearly dose-dependent and ranged from slowing-down cell proliferation rates and delay in development up to complete replication stop.

### Transgenic fish expressing Dmrt1bY show a delay in early development

**Figure 3:**
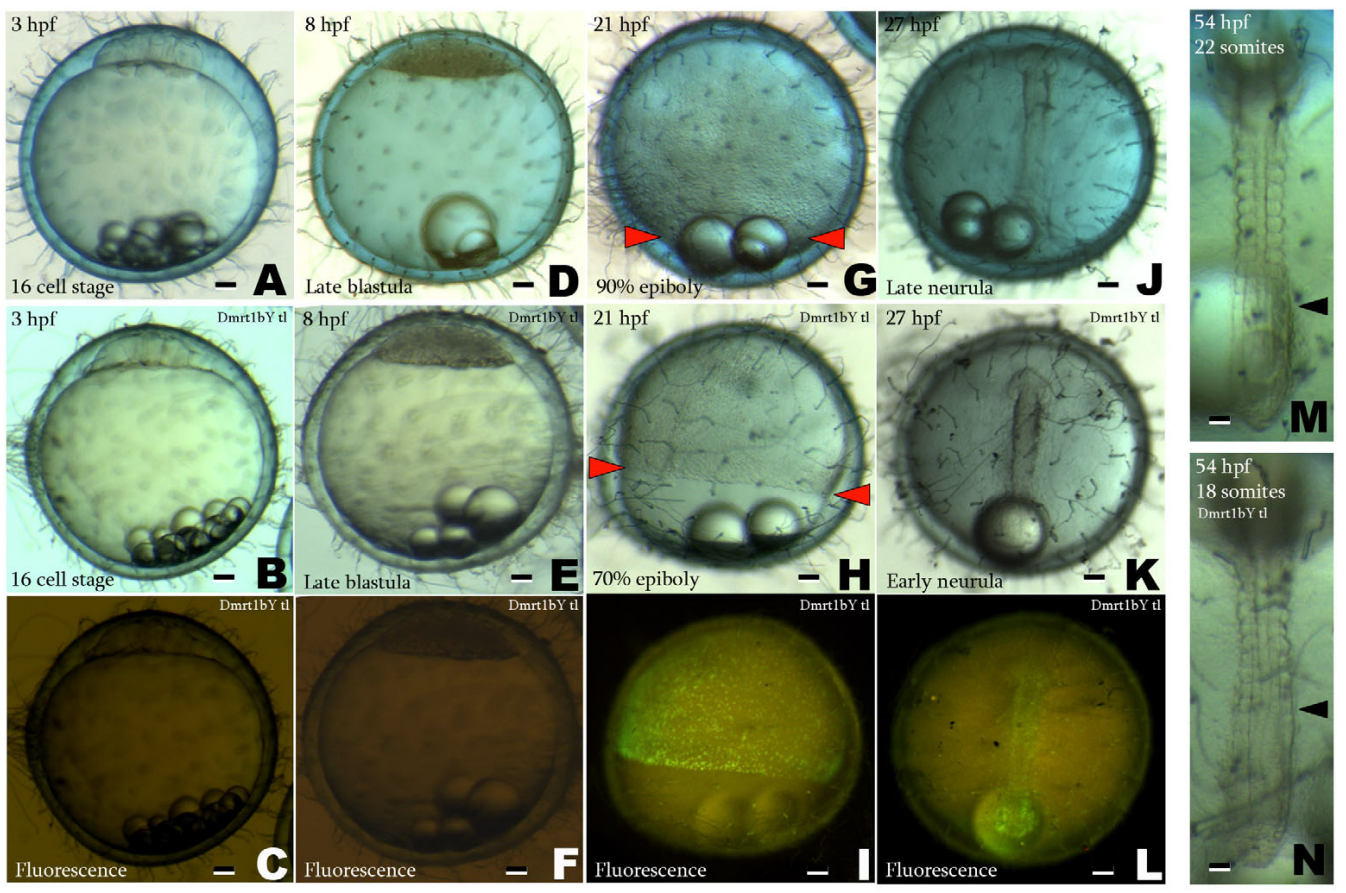
**Delay in early development in transgenic fish expressing Dmrt1bY under control of the cytoskeletal actin promoter**. A, D, G, J and M: normal wild type embryo at 16 cell stage, late blastula, 90% epiboly, late neurula and 22 somite stage corresponding to stages 6, 11, 16, 18 and 26 respectively. B, E, H, K and N: Dmrt1bY transgenic line embryo. C, F, I and L: GFP expression during early development of the Dmrt1bY transgenic line. Wild type and Dmrt1bY transgenic embryos at the same time of embryonic development were obtained in the same clutch of fertilized eggs by crossing a Dmrt1bY:GFP heterozygous parental fish to a non-transgenic fish. Scale bars: 100 μM in A to L and 50 μM in M and N.

As mRNA injection experiments can produce excessively high levels of a gene product in a transient way, we sought for a system that leads to more physiological levels of Dmrt1bY in early embryonic cells not interfering with normal development. A transgenic line expressing the Dmrt1bY:GFP fusion protein driven by the ubiquitously active cytoskeletal actin promoter was established and analysed for Dmrt1bY:GFP expression (Fig. 3). This transgenic line, however, does not show sex reversal, probably due to the ubiquitous rather than Sertoli-cell specific expression and generally low expression in later developmental stages of the *dmrt1bY *transgene.

**Figure 4:**
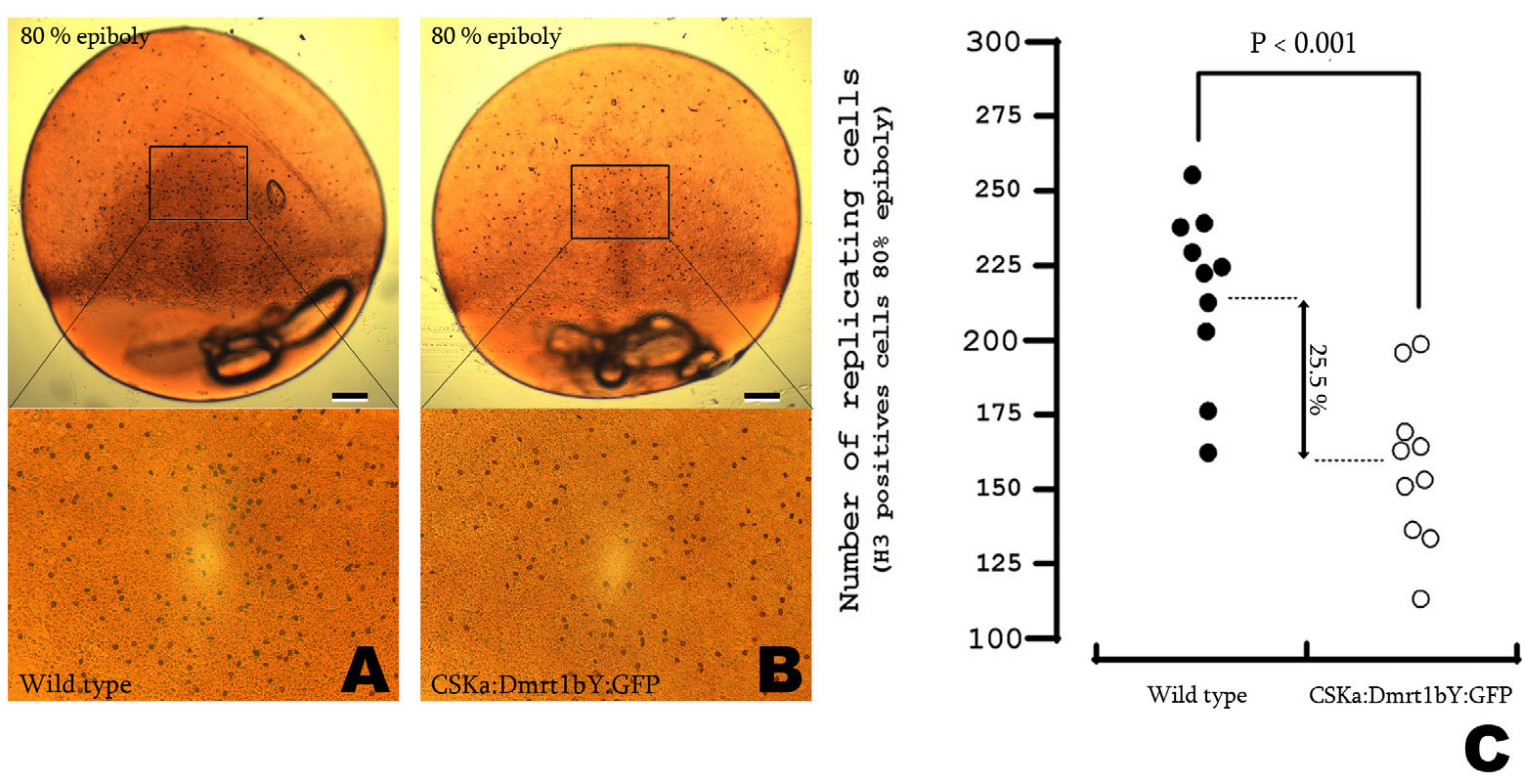
**Loss of replicating cells in transgenic fish expressing Dmrt1bY**. A, B and C: Cell replication status was quantified at 80% epiboly (stage 17) by pH3 immunostaining. Wild type (A), CSKA:Dmrt1bY:GFP transgenic line. pH3 positive cells were counted for a constant given area (A, B) and revealed a highly significant (p < 0.001) decrease of 25.5% replicating cells in the transgenic line when compared to wild type (C). Scale bars: 120 μM.

In embryos of the transgenic line, after zygotic transcription starts at mid-blastula (stage 11), a ubiquitous accumulation of the fusion protein was observed. During early neurulation (stage 17) GFP intensity reached its maximum. Interestingly this wave of Dmrt1bY:GFP protein expression was clearly paralleled by a marked delay in development for which the peak is also at early neurulation (stage 17) (Fig. 3). At the maximal Dmrt1bY:GFP protein accumulation at 70–80% epiboly (early neurulation, stage 17), cell replication status was quantified using pH3 staining (Fig. 4A,4B and 4C). While the spatial distribution of pH3 staining was comparable for both, wild type and transgenic embryos, a highly significant (p < 0.001) decrease of 25.5% in the number of replicating cells was observed for the Dmrt1bY:GFP protein expressing embryos (Fig. 4A and 4B). This value in the transgenics (Fig. 4C) is similar to the value obtained *in-vitro *in Dmrt1bY expressing cell lines (Fig. 1).

### Differences in primordial germ cell number, shape and replication pattern are diagnostic for the male and female gonad anlage

**Figure 5:**
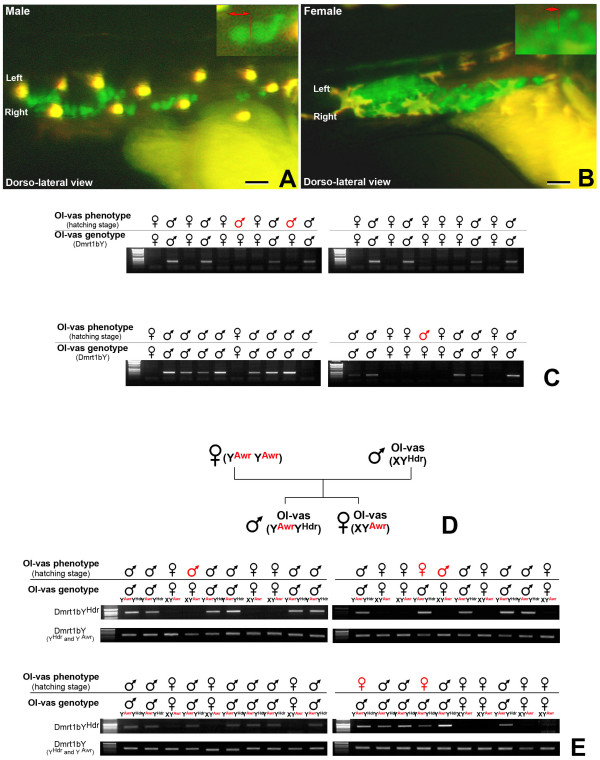
**Differences in primordial germ cell number and shape diagnostic for the male and female gonad primordium anlage**. A and B: Green fluorescence of the *Olvas *medaka primordial gonad at hatching stage (stage 39). Male (A), female (B). Both sexes have a significantly higher number of PGCs in the right PGC lateral clump. C: Comparison of the *Olvas *PGC phenotype versus genotype (Dmrt1bY) at hatching stage. D: Offspring obtained by mating a Y^Awr^/Y^Awr ^female with an Olvas X/Y^HdrR ^male. E: Comparison of the *Olvas *primordial gonad phenotype at hatching stage versus genotype (Y^Awr ^or Y^HdrR^). Identification of genetic sex in each individual was performed by PCR analysis for *dmrt1bY *presence or absence after genomic DNA extraction. The *dmrt1bY *fragments from either both Awr or Hd-rR-specific alleles were amplified with diagnostic primer sets. Scale bars: 7.5 μM.

Evidence has accumulated that the first appearance of morphological sex differentiation in medaka is a difference in the number of germ cells between the sexes [25,26]. Importantly, Dmrt1bY expression in the male gonad correlates with the appearance of male/female germ cell dimorphism. This first morphological sex difference is apparent as early as pre-hatching stage (stage 38) [18], more than ten days before the somatic gonad is formed [27]. Hence, taking advantage of the *Olvas *medaka transgenic line [28], which expresses GFP under control of the vasa promoter specifically in the primordial germ cells, we investigated the possibility of sexing medaka embryos as early as the hatching stage through primordial germ cell number and shape (Fig. 5A,5B and 5C).

**Figure 6:**
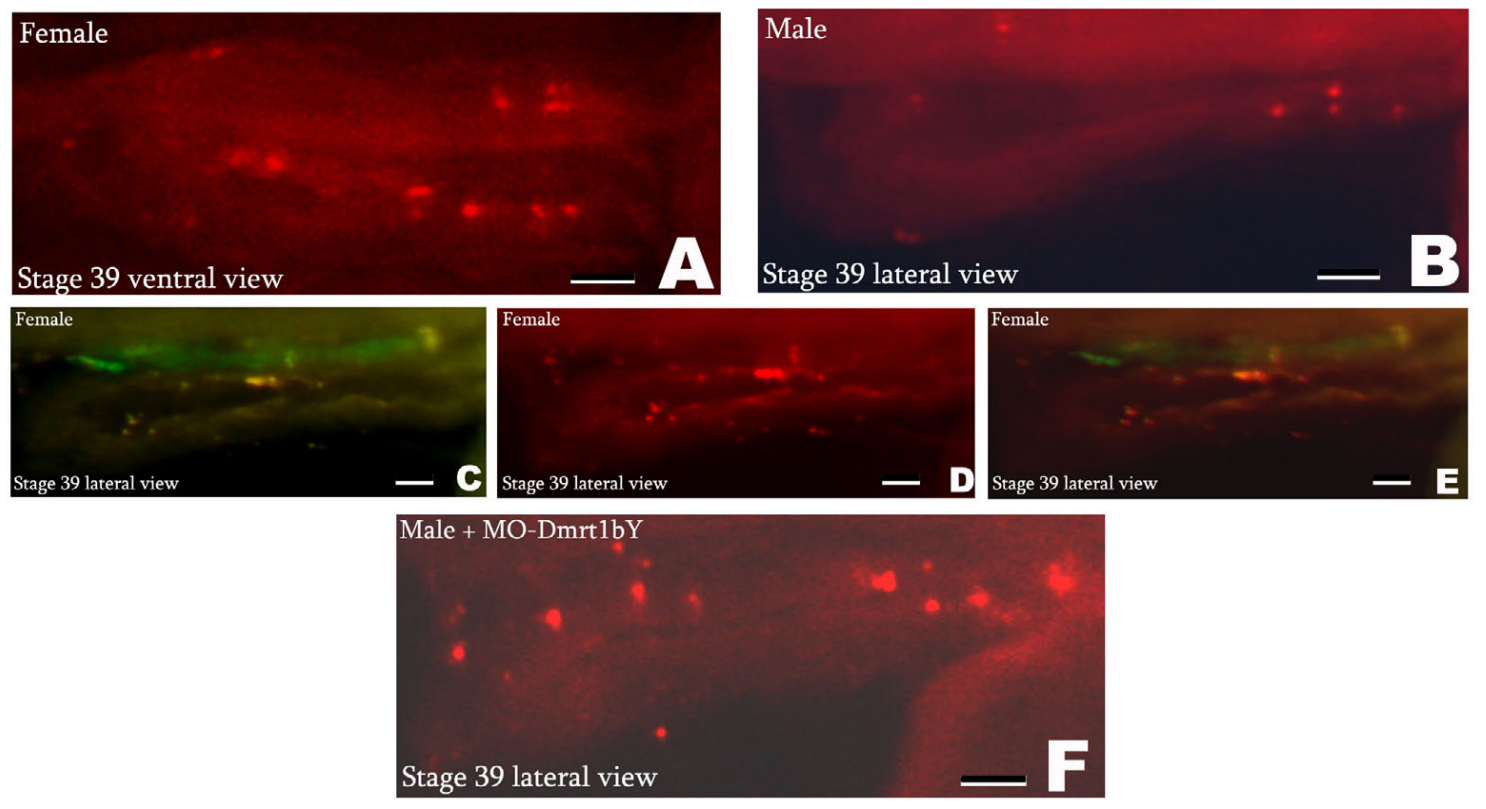
**Shift of male primordial gonad replication pattern towards female by down regulation of Dmrt1bY (BrdU incorporation)**. Replication pattern of the primordial gonad was followed by BrdU incorporation. A: female primordial gonad phenotype at hatching stage. B: male primordial gonad phenotype at hatching stage. C, D and E: fluorescence produced by BrdU incorporation colocalizes with GFP fluorescence of primordial germ cells in the Olvas transgenic line. GFP fluorescence (C), BrdU fluorescence (D), overlay (E). F: primordial gonad phenotype at hatching stage after Dmrt1bY morpholino injection in a genetic male.

Just after hatching (stage 39), GFP-positive PGC number in *Olvas *males was one half up to two third of *Olvas *females (40 to 70 and 100 to 150, respectively) (Fig. 5A and 5B). This is in accordance with the findings of Kobayashi et al. [18] and Hano et al. [29]. An additional criterion for discrimination was the shape of the area occupied by the PGCs. This materializes as a large ovoid cumulus-like cloud shape for females (Fig. 5B); while being thinner and more stretched (stratus-like cloud shape) along the anteroposterior axis for males (Fig. 5A). Noticeably, at stage 39, both sexes present a significant higher number of PGCs in the right PGC lateral clump, although this is more pronounced in females (Fig. 5A and 5B) [30]. Furthermore, individual primordial germ cells appear round and compact in males, while being smaller and more "diffuse" in females. Altogether these criteria allowed predicting sex of *Olvas *fish already shortly after hatching with a reliability of 92.5% (n = 40) (Fig. 5C). In addition, at stage 39 (hatching), BrdU incorporation assay revealed more replicating cells in female than in male (Fig. 6A and 6B). Noticeably, this enhanced cell replication in female was observed for both PGCs and the somatic surrounding cells (Fig. 6C,D and 6E).

In order to test whether the morphology of the developing gonad in *Olvas *transgenic fish correlates with expression of a functional *Dmrt1bY *gene, the Y^Awr ^mutant strain was used. In this strain *dmrt1bY *contains a nucleotide insertion in exon 3, resulting in a frame shift creating a premature stop at residue 139. Although this mutation is connected to a male to female sex reversal in XY^Awr ^and Y^Awr^Y^Awr ^fish [31,32], the underlying mechanism is unknown and sex-specific germ cell numbers and arrangement was not reported. Offspring obtained by mating a Y^Awr^Y^Awr ^female with an *Olvas *XY^HdrR ^male (Fig. 5D) containing the wild type *dmrt1bY *gene was analyzed according to the above mentioned criteria in order to determine sex by PGC and primordial gonad phenotypes. Presence of *dmrt1bY*^Awr ^and/or *dmrt1bY*^HdrR ^alleles was then assayed by PCR using specific oligonucleotide primers (Fig. 5E). This revealed that the PGC phenotype, like in wild type fish, is determined by the presence (male) or absence (female) of a functional *dmrt1bY*^HdrR ^gene (reliability = 87.5%, n = 40) (Fig. 5D and 5E).

### Down-regulation of Dmrt1bY shifts male primordial gonad phenotype towards female

**Figure 7:**
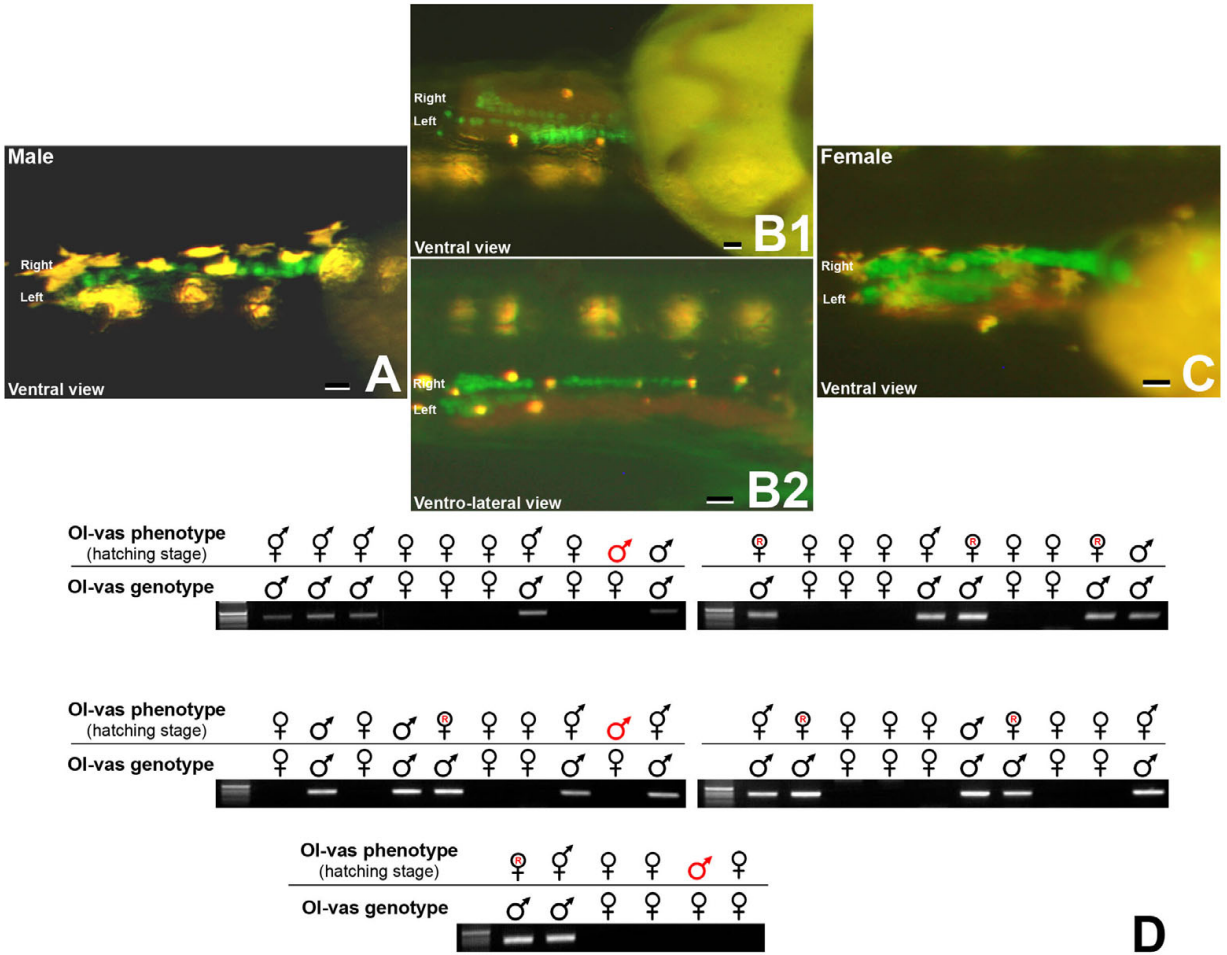
**Shift of male primordial gonad phenotype towards female by down regulation of Dmrt1bY**. A: Male primordial gonad phenotype at hatching stage (stage 39). B1 and B2: Partially shifted male to female primordial gonad phenotype at hatching stage after MO-Dmrt1bY morpholino injection. C: Female and totally reversed male primordial gonad phenotype at hatching stage after MO-Dmrt1bY morpholino injection. D: Comparison of the primordial gonad phenotype versus genotype after Dmrt1bY morpholino injection. Scale bars: 7.5 μM.

To this end the Dmrt1bY effect on cell proliferation could be demonstrated *in-vitro *as well as *in-vivo*. However, to verify that this function is of relevance for the gene's action in gonad determination and development we attempted to down-regulate *dmrt1bY *expression in developing embryos. Having the possibility of discriminating male and female primordial gonads by GFP phenotypes and BrdU incorporation at hatching stage, embryos were injected with a *dmrt1bY*-morpholino at the one cell stage and analyzed for gonad phenotype and genotype at hatching (stage 39) (Fig. 6 and 7).

In no case genotypic females were affected. In genotypic males (n = 40), Dmrt1bY morpholino injection resulted in mainly three phenotypes (Fig. 7A,7B1,7B2 and 7C). Complete primordial germ cell phenotypic sex reversion was observed for 17.4% of the males (Fig. 7C and 7D). Another 19.5% of the males exhibited an intermediate male/female PGC and gonad shape but made up of much more primordial germ cells when compared to wild type males (Fig. 7B1, 7B2 and 7D). The remaining 63.1% males showed no gross abnormalities (Fig. 7A). In injected embryos that were allowed to develop until adult stage, no sex reversion of the gonad was observed.

It should be noted that the effect of Dmrt1bY morpholino knockdown occurred exactly at the stage when dmrt1bY is first expressed in the pre-Sertoli cells and when the first difference in the presumptive male and female primordial gonad become visible. BrdU incorporation showed that in female and male-Dmrt1bY morpholino injected gonads, many PGCs were labelled (Fig. 6A and 6F) while in genotypically male control embryos a lower number of BrdU labelled cells were observed (Fig. 6B). The ratio between male and female BrdU-labelled gonadal cells was 0.1 – 0.2 in non injected fishes. In fish injected with Dmrt1bY morpholino this ratio increased to 0.3 – 0.4, indicative of higher proliferation rate. Similar results were obtained when checking at cell replication using phospho-histone H3 staining [see additional file 2]. Interestingly, in female and male-Dmrt1bY morpholino injected embryos, the higher number of labelled PGCs was paralleled with a higher replication status of the surrounding gonad mesoderm (Fig. 6C,D and 6E).

## Discussion

From nematodes to mammals, evidence has accumulated that *dmrt1 *genes are involved in sex determination [9,19,32-34]. However, nothing is known how these genes exert their function during gonad induction, differentiation and maintenance in vertebrates. Considering the fact that in medaka like in many other fish [35] the first sign of sexual differentiation is a lower number of PGCs in male embryos has led to the obvious hypothesis that Dmrt1bY regulates PGC numbers at the hatching stage in males ([36] for review). A lower number of PGCs in the gonad primordium of males could be due to a negative control of proliferation at one of the various check points of the cell cycle, reduction of PGCs by apoptosis or phagocytosis, or loss of PGCs through migration out of the gonad primordium. Our results support the view that medaka Dmrt1bY negatively regulates cell proliferation through an arrest in the G2 phase. Graded ectopic expression of Dmrt1bY correlated with the degree of down-regulation of cell proliferation, up to complete arrest. Apoptosis appeared to be a late consequence of Dmrt1bY toxicity. However, the molecular mechanisms by which medaka Dmrt1bY exerts its effect remain to be determined.

Dmrt1bY effects on cell proliferation in embryos were only observed, when zygotic transcription had already started after MBT. Combined with the apparent nuclear localization this opens the possibility that Dmrt1bY may be involved in regulation of genes that control cell proliferation. However, Dmrt1 transcriptional activity has not been demonstrated in vertebrates yet, but has been established for the *Drosophila doublesex *and *C. elegans mab-3 *orthologues of *Dmrt1 *[8,37,38].

Medaka Dmrt1bY is exclusively expressed in pre-Sertoli cells of the male primordial gonad at hatching stage as well as in adult testes in mature Sertoli cells [18]. Thus, it is possible that one early function of Dmrt1bY is to negatively regulate cell proliferation in the Sertoli cell lineage. This would be responsible for Sertoli cell quiescence, which is a hallmark of this cell type. Such hypothesis is supported by the only other functional study on a *dmrt1 *orthologue from vertebrates: in *dmrt1*-/- mutant mice Sertoli cells show an abundant overproliferation [19].

Beside an obvious cell autonomous effect on the cell cycle, also a non-cell autonomous, possibly juxtacrine action was observed. This juxtacrine action correlates with the observations of Shinomiya [27] showing that in Medaka in XX/XY transplantation chimeras, XY somatic cells differentiate into male cells according to their sex chromosome composition and that in this environment XX germ cells differentiate into male cells.

We have shown that already 10 days before somatic gonadal dimorphism, sex can be diagnosed through both primordial germ cell phenotype and somatic gonad replication rate just after hatching in live embryos. At this stage the number of primordial germ cells is higher in XX embryos than that of XY embryos [18,21]. Dmrt1bY has been shown to be exclusively expressed in the PGC supporting pre-Sertoli cells [18]. Hence, during primordial gonad formation, Dmrt1bY primary function could be to regulate both supporting cell replication and primordial germ cell proliferation *via *a non-cell autonomous mechanism. We have shown here that Dmrt1bY can act in a juxtacrine way to downregulate proliferation and that Dmrt1bY-morpholino injection led to modulation of the early "primordial gonad" phenotype, transiently shifting male primordial germ/supporting cell phenotype towards the female state. The transient nature of the shift from male to female PGC phenotype at hatching stage after Dmrt1bY morpholino injection is due to the limited stability of the morpholino and clearly indicates that Dmrt1bY action during male primordial gonad commitment is not strictly inductive but requires a prolonged expression.

In medaka it has been demonstrated that XY supporting somatic cells can initiate sex differentiation into the male type regardless of the presence of coexisting germ cells [39]. In addition, cell transplantations between blastula embryos in medaka suggest that XY somatic cells produce an environment that leads to the differentiation of primordial germ cells into male germ cells regardless of their sex chromosome composition [27]. In this context, two main functions can be concomitantly attributed to the master sex determining *dmrt1bY *gene in testis development in medaka. First, because from embryo to adult, continuous Dmrt1bY expression occurs specifically in the Sertoli cell lineage [18] and cell autonomous differentiation of Sertoli cell differentiation has been observed in XY gonads lacking germ cells [39], Dmrt1bY is likely to induce the development and the maintenance of the Sertoli cell lineage in an autocrine manner like its mammalian counterpart *Sry*. Second, Dmrt1bY appears to be responsible for the male primordial germ cell arrest from the pre-hatching stage (stage 38–39) until 20–30 days after hatching, likely *via *a juxtacrine mechanism.

## Conclusion

The finding that Dmrt1bY downregulates primordial germ cell proliferation already 10 days before somatic gonadal differentiation, leads us to suggest that in XY medaka males, Dmrt1bY driven PGC number regulation as well as determination of pre-Sertoli cells is the primary event by which the whole gonad (germ line and soma) would be specified through a directional cross talk from pre-Sertoli/Sertoli cells with PGCs. Then, at this stage, Sertoli cells would be inhibiting primordial germ cell proliferation in the male primordial gonad through juxtacrine Dmrt1bY action.

## Methods

### Fish maintenance and breeding

Medaka were taken from closed breeding stocks of the Carbio (Carolina Biological Supplies) strain and kept under standard conditions. Medaka embryos were staged according to Iwamatsu [40].

### Immunochemistry, DAPI staining and proliferation quantification

For antibody staining, embryos were staged and fixed overnight in 4% paraformaldehyde (PFA) in phosphate buffered saline (PBS) at 4°C. After washing and dechorionation, they were dehydrated and stored in methanol at -20°C. After stepwise rehydration in PBS embryos were incubated in blocking solution (1% DMSO, 1% BSA, 1% goat serum and 0.5% Triton X100 in PBS) for 1 hour at room temperature. Anti-phospho-Histone3 antibody (Upstate) was prepared in blocking solution at 1:100 dilution. Incubation with antibody was performed overnight at 4°C. After extensive washes in PBS, embryos were incubated with a 1:200 dilution of goat anti-rabbit horseradish peroxidase (HRP) conjugated secondary antibody in 1% bovine serum albumin (BSA) in PBS overnight at 4°C. Embryos were then washed 3 times in PBS and the colour reaction was developed using the ABC kit (Sigma) with horseradish peroxidase and DAB as chromogen. Ten individuals derived from the same parents were examined at 80% epiboly (stage 17). Mean and SEM of H3 positive cells were calculated. Differences between wild type and transgenic fish were evaluated statistically by paired Student test.

Nuclear staining was performed with DAPI (4,6-diamidino-2-phenylindole, 1 μg/mL final concentration) for 30 minutes and observed under a fluorescent microscope.

For the identification of proliferating cells that had passed through S phase we used the 5-bromo-2'-deoxyuridine (BrdU) labelling technique. For BrdU labelling, embryos were incubated for 10–12 hours in 10 mM BrdU (sigma) and 2% tween 20 in solution to permeabilize embryos. Proliferating cells which had incorporated BrdU were identified using specific antibodies coupled with Alexa and observed under fluorescent microscope.

### Cell death assay

TUNEL (terminal transferase mediated dUTP nick end-labeling) assay (ApopTag Peroxidase In situ kit, Chemicon) was used to assess apoptosis in wild type, *dmrt1bY *RNA injected and transgenic fish embryos. Embryos were staged and fixed overnight in 4% PFA in PBS at 4°C, then washed in PBS/0.1% Tween 20 (PBT) and dechorionated, dehydrated stepwise to methanol, and stored at -20°C. After rehydration, embryos were permeabilized by proteinase K digestion, re-fixed in buffered 4% PFA, washed in PBT and subsequently placed in equilibration buffer according to manufacturer's instructions. After one hour incubation at room temperature, equilibration buffer was removed and embryos were then placed for one hour at 37°C in digoxigenin-labeled dNTP containing reaction buffer and terminal transferase enzyme. Next, embryos were extensively washed in stop/wash buffer and then 3 times in PBT and incubated overnight at 4°C in anti-digoxigenin peroxidase conjugate containing buffer. After 4 washes in PBT, digoxigenin-labeled dNTP incorporation was detected with the peroxidase ABC Kit using DAB as chromogen.

### Production of transgenic fish

For generation of transgenic lines, the meganuclease system according to Thermes et al., 2002 was used. The *I-SceI-pCSKa::Dmrt1bY::GFP *plasmid was obtained by replacing the cytoskeletal actin promoter driven reporter cassette of *pCSKaGFPS-I *vector [41] with the *Dmrt1bY:GFP *construct. Briefly, *Dmrt1bY::GFP *fragment was obtained by cloning *Dmrt1bY *complete ORF [4] (*EcoRI/BamHI *sites) into pEGFP-N1 plasmid. The resulting plasmid was cut with *EcoRI/NotI *and the *Dmrt1bY::GFP *fragment was inserted into I-SceI-pCSKa plasmid (*EcoRI/NotI *sites). Plasmid DNA was injected at a concentration of 100 ng/μl together with the *I-SceI *meganuclease enzyme (0.5 unit/μL in 1 X *I-SceI *buffer), through the chorion into the cytoplasm of the one-cell embryo. Embryos were kept at 28.5°C until hatching. GFP-expressing F0 fish were selected as putative founder fish, raised to sexual maturity, mated to wild type partners and tested for germline transmission. Sex ratio was not affected.

### RNA and morpholino injections

For RNA injection, *Dmrt1bY *and *ΔDmrt1bY *fused to GFP were cloned into pCS2 plasmid (*EcoRI/NotI *sites). Deletion of DM domain for *ΔDmrt1bY *construct was achieved by removing nucleotides 28 to 232 of the Dmrt1bY ORF after *PpuMI/StuI *digestion and religation.

Capped RNA for injections was transcribed from linearized vectors using the SP6/T3/T7 m MESSAGE mMACHINE Kit (Ambion). One nL was injected into the cytoplasm of one-cell stage Medaka embryos as described [42]. For knockdown experiments, embryos were injected with the morpholino RT1Y: 5'-TCAGACAAAAACATCCAAATCCAGT-3' directed against the Dmrt1bY 5' UTR. The most efficient morpholino dose (450 ng/μl) was experimentally determined and the specificity of the oligo confirmed in control experiments. The non-overlapping expression pattern observed for Dmrt1bY and Dmrt1a transcripts during embryogenesis between hatching stage up to 5 days after hatching [4,5,35,43] rules out a possible cross-reactivity of the Dmrt1bY morpholino against Dmrt1a. In addition, the morpholino target sequence in dmrt1bY has 5 mismatches to the corresponding sequence in dmrt1a [see additional file 3].

### Determination of phenotypic and genotypic sex in Olvas-GFP medaka

The *Olvas*-*GFP *medaka line [28] was provided by M. Tanaka. Homozygous *Olvas-GFP *fish were crossed either with Hd-rR or Awr strain fish [5,44]. Phenotypic sex was determined according to both, number [18,26,29] and shape of primordial germ cells (GFP positive cells) as well as shape of the primordial gonad at stage 39 (day of hatching). Subsequently, identification of genetic sex in each individual was performed by PCR analysis for *dmrt1bY *presence or absence after genomic DNA extraction. The *dmrt1bY *fragments from either both Awr or Hd-rR-specific alleles were amplified with diagnostic primer sets, DMTYa: 5'-GGCCGGGTCCCCGGGTG-3'/DMTYd: 5'-TTTGGGTGAACTCACATGG-3' and DMTYe: 5'-ACAGGTAAACCAGAAAAACTA-3'/DMT1l: 5'-AACTAATTCATCCCCATTCC-3' respectively. Phenotypic sexing reliability (%) through primordial germ cell visualization was calculated by comparing GFP-primordial gonad phenotypes with genotypes (presence or absence of functional *dmrt1bY*).

### Cell culture, transfection and flow cytometric analysis

Mouse Sertoli TM4 cells and *Xiphophorus *embryonic epithelial A2 cells were cultured as described by Beverdam et al. [23] and by Kuhn et al. [45] respectively. Cells were grown to 70–80% confluence in 6-well plates and then transfected with 5 μg expression vector using GeneJuice reagent (Novagen) as described by the manufacturer. Cell suspension of *Dmrt1bY *mRNA-injected and control embryos was achieved by manual dechorionation at stage 13. Cells were then fixed in 80% ethanol overnight at -20°C and then stained with propidium iodide (15 μg/mL). Native cells were stained with Hoechst dye 33342 at a final concentration of 15 microg/mL (by dilution from trihydrochloride trihydrate stock at 10 mg/mL, Molecular Probes, Eugene, OR) at 37 °C in the dark. Immediately before measurement of a sample, propidium iodide (PI) was added, likewise at a final concentration of 15 μg/mL (by dilution from stock at 1 mg/mL, Molecular Probes, Eugene, OR). Univariate flow histograms were recorded on an analytical, dual-laser equipped flow cytometer (LSR, Becton Dickinson, Heidelberg, Germany) using UV excitation of the Hoechst dye and gating on vital cell via PI exclusion. The resulting cell cycle distributions reflected by DNA content were quantitated using the MPLUS AV software package (Phoenix Flow Systems, San Diego, CA). All transfection/cell cycle experiments were repeated three times and gave similar results. [data are shown in additional file 1].

## Authors' contributions

AH carried out the embryological, cell biological and molecular genetic studies, and drafted the manuscript. DS carried out the flow cytometric determinations and did the analysis of the cell cycle experiments. AK did the cell culture experiments. UH did the cloning and sequencing of the dmrt1 expression constructs and produced the transgenic fish line. CW showed the specificity of the morpholino and did some control injections. AH and MS conceived the study. MS supervised the crossing experiments and the embryo production. MS participated in the design and coordinated the experiments and wrote the manuscript. All figures were prepared by AH. All authors read and approved the final manuscript.

## Supplementary Material

Additional file 1 **Dmrt1bY overexpression modifies cell cycle pattern in cell culture as well as in live embryos**. A and B: Radar histograms representing the DNA content distribution of Dmrt1bY:GFP and control GFP transfected cells. Raw data are expressed in percentage of cells in either G1, S or G2 phase. C: Cell cycle distribution reflected by DNA content in control *versus *stage 10 (just MBT) and post-MBT (stage 13) *Dmrt1bY*-injected embryos. D: Cell cycle distribution reflected by DNA content variation in *Xiphophorus *embryonic epithelial A2 cells, represented in percentage of the control in control and Dmrt1bY expressing cells. Click here for file

Additional file 2 **Analysis of apoptosis in Dmrt1bY overexpressing embryos**. A, B, C, D, E and F: Cell death assay. TUNEL assay was used to assess apoptosis in wild type (A, C and E) and in Dmrt1bY injected (B, D and F) embryos. G to N: Increased apoptosis in transgenic fish expressing Dmrt1bY. TUNEL assay was used to investigate apoptosis in wild type (G, H, I and J) and in CSKa:Dmrt1bY:GFP transgenic line (K, L, M and N). Click here for file

Additional file 3**Analysis of Morpholino efficiency and specificity**. 25 pg of a construct containing 557 bp of the upstream DMRT1bY region including UTR and ATG, which drives a GFP reporter (fused in-frame with a DMRT1a cDNA), were injected alone or in combination with 0.85 ng DMRT1bY Morpholino into Medaka embryos at the one to two cell stage. Injection of the DNA alone resulted in strong GFP expression (in 11/12 embryos; arrows in A), which was significantly reduced in embryos coinjected with the DMRT1bY Morpholino (weak GFP expression in 15/22 embryos). GFP expression was not reduced, when a control Morpholino (directed against the autosomal copy of DMRT1) containing five base pair changes compared to the DMRT1bY sequence was coinjected with the DMRT1Y-GFP fusion construct (strong GFP expression in 15/15 embryos; arrows in C). Higher magnification views in (D-F) show nuclear GFP expression, which was predominantly found in the marginal zone of embryos analyzed at 50% epiboly. Comparison of the dmrt1bY specific morpholino sequence to the corresponding sequence in dmrt1a: Click here for file

## References

[B1] Raymond CS, Parker ED, Kettlewell JR, Brown LG, Page DC, Kusz K, Jaruzelska J, Reinberg Y, Flejter WL, Bardwell VJ, Hirsch B, Zarkower D (1999). A region of human chromosome 9p required for testis development contains two genes related to known sexual regulators. Hum Mol Genet.

[B2] Nanda I, Shan Z, Schartl M, Burt DW, Koehler M, Nothwang H, Grutzner F, Paton IR, Windsor D, Dunn I, Engel W, Staeheli P, Mizuno S, Haaf T, Schmid M (1999). 300 million years of conserved synteny between chicken Z and human chromosome 9. Nat Genet.

[B3] Shetty S, Kirby P, Zarkower D, Graves JA (2002). DMRT1 in a ratite bird: evidence for a role in sex determination and discovery of a putative regulatory element. Cytogenet Genome Res.

[B4] Nanda I, Kondo M, Hornung U, Asakawa S, Winkler C, Shimizu A, Shan Z, Haaf T, Shimizu N, Shima A, Schmid M, Schartl M (2002). A duplicated copy of DMRT1 in the sex-determining region of the Y chromosome of the medaka, Oryzias latipes. Proc Natl Acad Sci USA.

[B5] Matsuda M, Nagahama Y, Shinomiya A, Sato T, Matsuda C, Kobayashi T, Morrey CE, Shibata N, Asakawa S, Shimizu N, Hori H, Hamaguchi S, Sakaizumi M (2002). DMY is a Y-specific DM-domain gene required for male development in the medaka fish. Nature.

[B6] Zhu L, Wilken J, Phillips NB, Narendra U, Chan G, Stratton SM, Kent SB, Weiss MA (2000). Sexual dimorphism in diverse metazoans is regulated by a novel class of intertwined zinc fingers. Genes Dev.

[B7] An W, Wensink PC (1995). Three protein binding sites form an enhancer that regulates sex- and fat body-specific transcription of Drosophila yolk protein genes. Embo J.

[B8] Yi W, Zarkower D (1999). Similarity of DNA binding and transcriptional regulation by Caenorhabditis elegans MAB-3 and Drosophila melanogaster DSX suggests conservation of sex determining mechanisms. Development.

[B9] Yi W, Ross JM, Zarkower D (2000). Mab-3 is a direct tra-1 target gene regulating diverse aspects of C. elegans male sexual development and behavior. Development.

[B10] Baker BS, Ridge KA (1980). Sex and the single cell. I. On the action of major loci affecting sex determination in Drosophila melanogaster. Genetics.

[B11] Christiansen AE, Keisman EL, Ahmad SM, Baker BS (2002). Sex comes in from the cold: the integration of sex and pattern. Trends Genet.

[B12] Winkler C, Hornung U, Kondo M, Neuner C, Duschl J, Shima A, Schartl M (2004). Developmentally regulated and non-sex-specific expression of autosomal dmrt genes in embryos of the Medaka fish (Oryzias latipes). Mech Dev.

[B13] Raymond CS, Kettlewell JR, Hirsch B, Bardwell VJ, Zarkower D (1999). Expression of Dmrt1 in the genital ridge of mouse and chicken embryos suggests a role in vertebrate sexual development. Dev Biol.

[B14] Smith CA, McClive PJ, Western PS, Reed KJ, Sinclair AH (1999). Conservation of a sex-determining gene. Nature.

[B15] Kettlewell JR, Raymond CS, Zarkower D (2000). Temperature-dependent expression of turtle Dmrt1 prior to sexual differentiation [letter]. Genesis.

[B16] Marchand O, Govoroun M, D'Cotta H, McMeel O, Lareyre J, Bernot A, Laudet V, Guiguen Y (2000). DMRT1 expression during gonadal differentiation and spermatogenesis in the rainbow trout, Oncorhynchus mykiss. Biochim Biophys Acta.

[B17] Aoyama S, Shibata K, Tokunaga S, Takase M, Matsui K, Nakamura M (2003). Expression of Dmrt1 protein in developing and in sex-reversed gonads of amphibians. Cytogenet Genome Res.

[B18] Kobayashi T, Matsuda M, Kajiura-Kobayashi H, Suzuki A, Saito N, Nakamoto M, Shibata N, Nagahama Y (2004). Two DM domain genes, DMY and DMRT1, involved in testicular differentiation and development in the medaka, Oryzias latipes. Dev Dyn.

[B19] Raymond CS, Murphy MW, O'Sullivan MG, Bardwell VJ, Zarkower D (2000). Dmrt1, a gene related to worm and fly sexual regulators, is required for mammalian testis differentiation. Genes Dev.

[B20] Zhang J (2004). Evolution of DMY, a newly emergent male sex-determination gene of medaka fish. Genetics.

[B21] Satoh N, Egami N (1972). Sex differentiation of germ cells in the teleost, Oryzias latipes, during noraml embryonic development. J Embryol Exp Morphol.

[B22] Paul-Prasanth B, Matsuda M, Lau EL, Suzuki A, Sakai F, Kobayashi T, Nagahama Y (2006). Knock-down of DMY initiates female pathway in the genetic male medaka, Oryzias latipes. Biochem Biophys Res Commun.

[B23] Beverdam A, Wilhelm D, Koopman P (2003). Molecular characterization of three gonad cell lines. Cytogenet Genome Res.

[B24] Zhang L, Hua Z, Ren J, Meng A (2001). The nuclear localization signal of zebrafish terra is located within the DM domain. FEBS Lett.

[B25] Carter AD, Sible JC (2003). Loss of XChk1 function triggers apoptosis after the midblastula transition in Xenopus laevis embryos. Mech Dev.

[B26] Quirk JG, Hamilton JB (1973). Number of germ cells in known male and known female genotypes of vertebrate embryos (Oryzias latipes). Science.

[B27] Shinomiya A, Shibata N, Sakaizumi M, Hamaguchi S (2002). Sex reversal of genetic females (XX) induced by the transplantation of XY somatic cells in the medaka, Oryzias latipes. Int J Dev Biol.

[B28] Tanaka M, Kinoshita M, Kobayashi D, Nagahama Y (2001). Establishment of medaka (Oryzias latipes) transgenic lines with the expression of green fluorescent protein fluorescence exclusively in germ cells: a useful model to monitor germ cells in a live vertebrate. Proc Natl Acad Sci USA.

[B29] Hano T, Oshima Y, Oe T, Kinoshita M, Tanaka M, Wakamatsu Y, Ozato K, Honjo T (2005). Quantitative bio-imaging analysis for evaluation of sexual differentiation in germ cells of olvas-GFP/ST-II YI medaka (Oryzias latipes) nanoinjected in ovo with ethinylestradiol. Environ Toxicol Chem.

[B30] Hamaguchi S (1996). Bilaterally asymetrical testes in fishes of the genus Oryzias. Zoolog Sci.

[B31] Otake H, Shinomiya A, Matsuda M, Hamaguchi S, Sakaizumi M (2006). Wild-derived XY sex-reversal mutants in the Medaka, Oryzias latipes. Genetics.

[B32] Schartl M (2004). A comparative view on sex determination in medaka. Mech Dev.

[B33] Shen MM, Hodgkin J (1988). mab-3, a gene required for sex-specific yolk protein expression and a male-specific lineage in C. elegans. Cell.

[B34] Burtis KC, Baker BS (1989). Drosophila doublesex gene controls somatic sexual differentiation by producing alternatively spliced mRNAs encoding related sex-specific polypeptides. Cell.

[B35] Devlin RH, Nagahama Y (2002). Sex determination and sex differentiation in fish: an overview of genetic, physiological, and environmental influences. Aquaculture.

[B36] Matsuda M (2005). Sex determination in the teleost medaka, Oryzias latipes. Annu Rev Genet.

[B37] Ross JM, Kalis AK, Murphy MW, Zarkower D (2005). The DM domain protein MAB-3 promotes sex-specific neurogenesis in C. elegans by regulating bHLH proteins. Dev Cell.

[B38] Burtis KC, Coschigano KT, Baker BS, Wensink PC (1991). The doublesex proteins of Drosophila melanogaster bind directly to a sex-specific yolk protein gene enhancer. Embo J.

[B39] Shinomiya A, Hamaguchi S, Shibata N (2001). Sexual differentiation of germ cell deficient gonads in the medaka, Oryzias latipes. J Exp Zool.

[B40] Iwamatsu T (2004). Stages of normal development in the medaka Oryzias latipes. Mech Dev.

[B41] Thermes V, Grabher C, Ristoratore F, Bourrat F, Choulika A, Wittbrodt J, Joly JS (2002). I-SceI meganuclease mediates highly efficient transgenesis in fish. Mech Dev.

[B42] Koster R, Stick R, Loosli F, Wittbrodt J (1997). Medaka spalt acts as a target gene of hedgehog signaling. Development.

[B43] Hornung U, Herpin A, Schartl M (2007). Expression of the male determining gene dmrt1bY and its autosomal coorthologue dmrt1a in Medaka. Sex Dev.

[B44] Shinomiya A, Otake H, Togashi K, Hamaguchi S, Sakaizumi M (2004). Field survey of sex-reversals in the medaka, Oryzias latipes: genotypic sexing of wild populations. Zoolog Sci.

[B45] Kuhn C, Vielkind U, Anders F (1979). Cell cultures derived from embryos and melanoma of poeciliid fish. In Vitro.

