# Supplementary figures and images for: Inhibition of primordial germ cell proliferation by the medaka male determining gene Dmrt1bY
Source: BMC Dev Biol. 2007 Aug 30;7:99. doi: 10.1186/1471-213X-7-99 (PMC2034567; doi:10.1186/1471-213X-7-99)

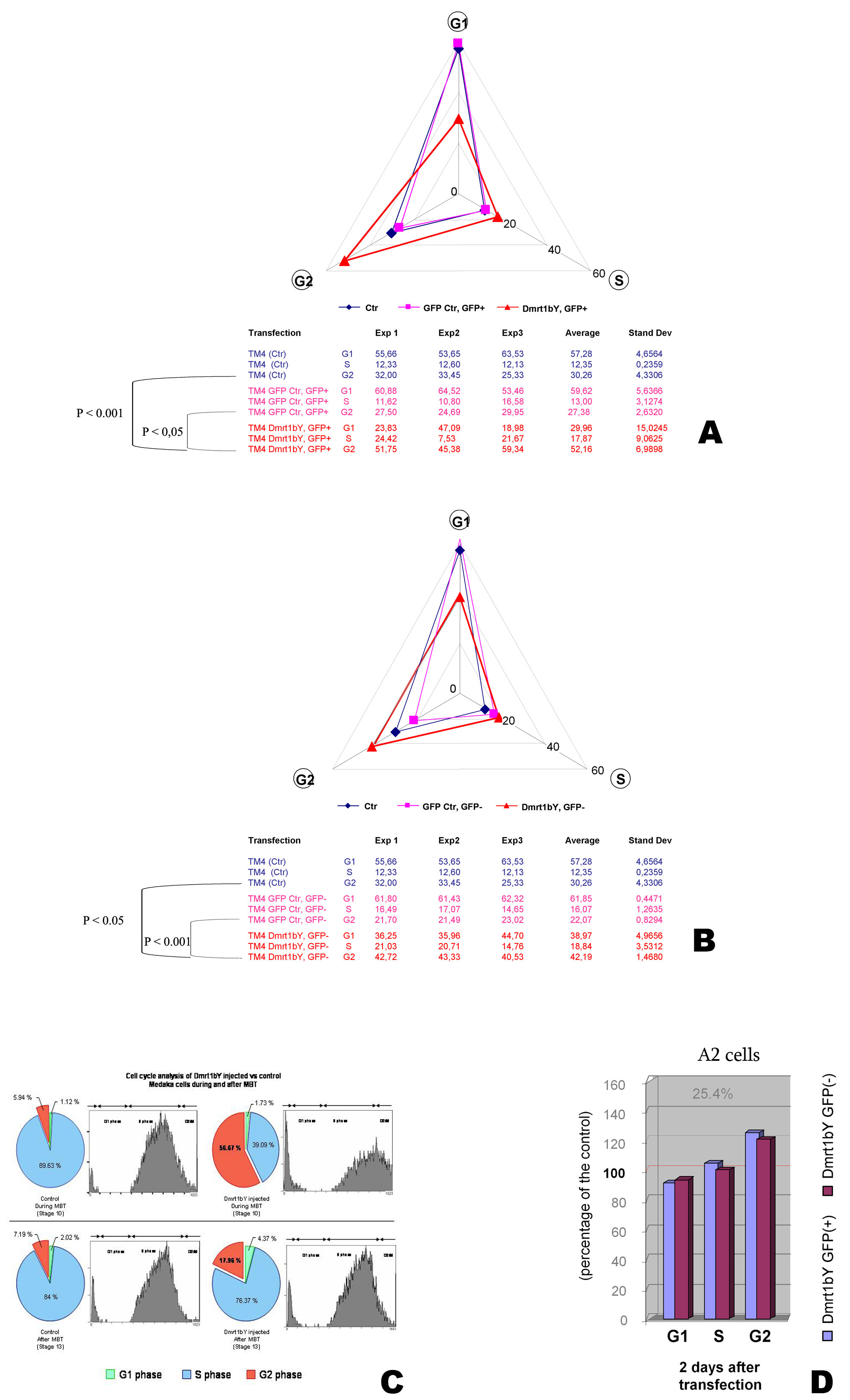

Supplement: Additional file 1 — Dmrt1bY overexpression modifies cell cycle pattern in cell culture as well as in live embryos. A and B: Radar histograms representing the DNA content distribution of Dmrt1bY:GFP and control GFP transfected cells. Raw data are expressed in percentage of cells in either G1, S or G2 phase. C: Cell cycle distribution reflected by DNA content in control versus stage 10 (just MBT) and post-MBT (stage 13) Dmrt1bY-injected embryos. D: Cell cycle distribution reflected by DNA content variation in Xiphophorus embryonic epithelial A2 cells, represented in percentage of the control in control and Dmrt1bY expressing cells. [file 1471-213X-7-99-S1.jpeg]

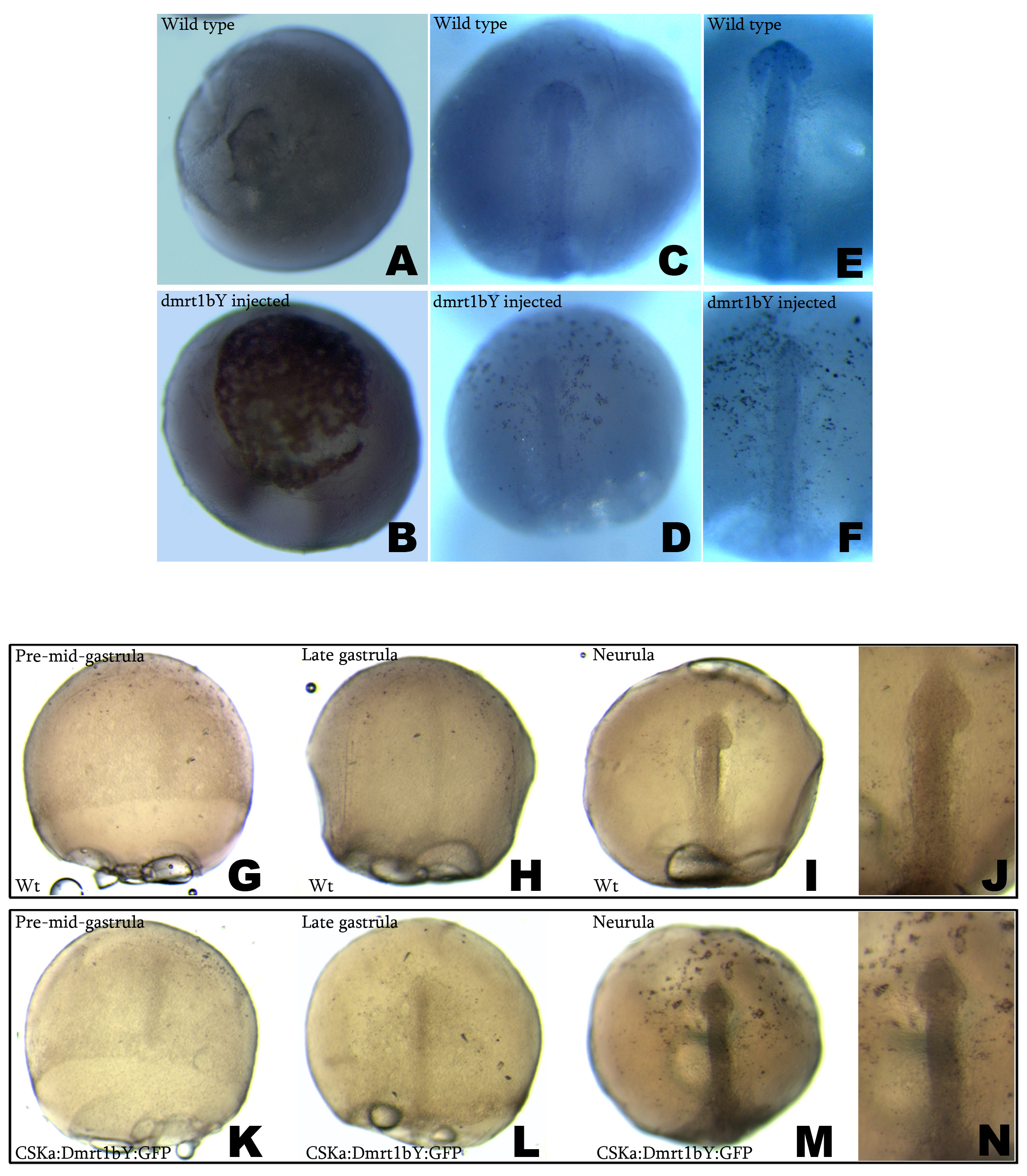

Supplement: Additional file 2 — Analysis of apoptosis in Dmrt1bY overexpressing embryos. A, B, C, D, E and F: Cell death assay. TUNEL assay was used to assess apoptosis in wild type (A, C and E) and in Dmrt1bY injected (B, D and F) embryos. G to N: Increased apoptosis in transgenic fish expressing Dmrt1bY. TUNEL assay was used to investigate apoptosis in wild type (G, H, I and J) and in CSKa:Dmrt1bY:GFP transgenic line (K, L, M and N). [file 1471-213X-7-99-S2.jpeg]

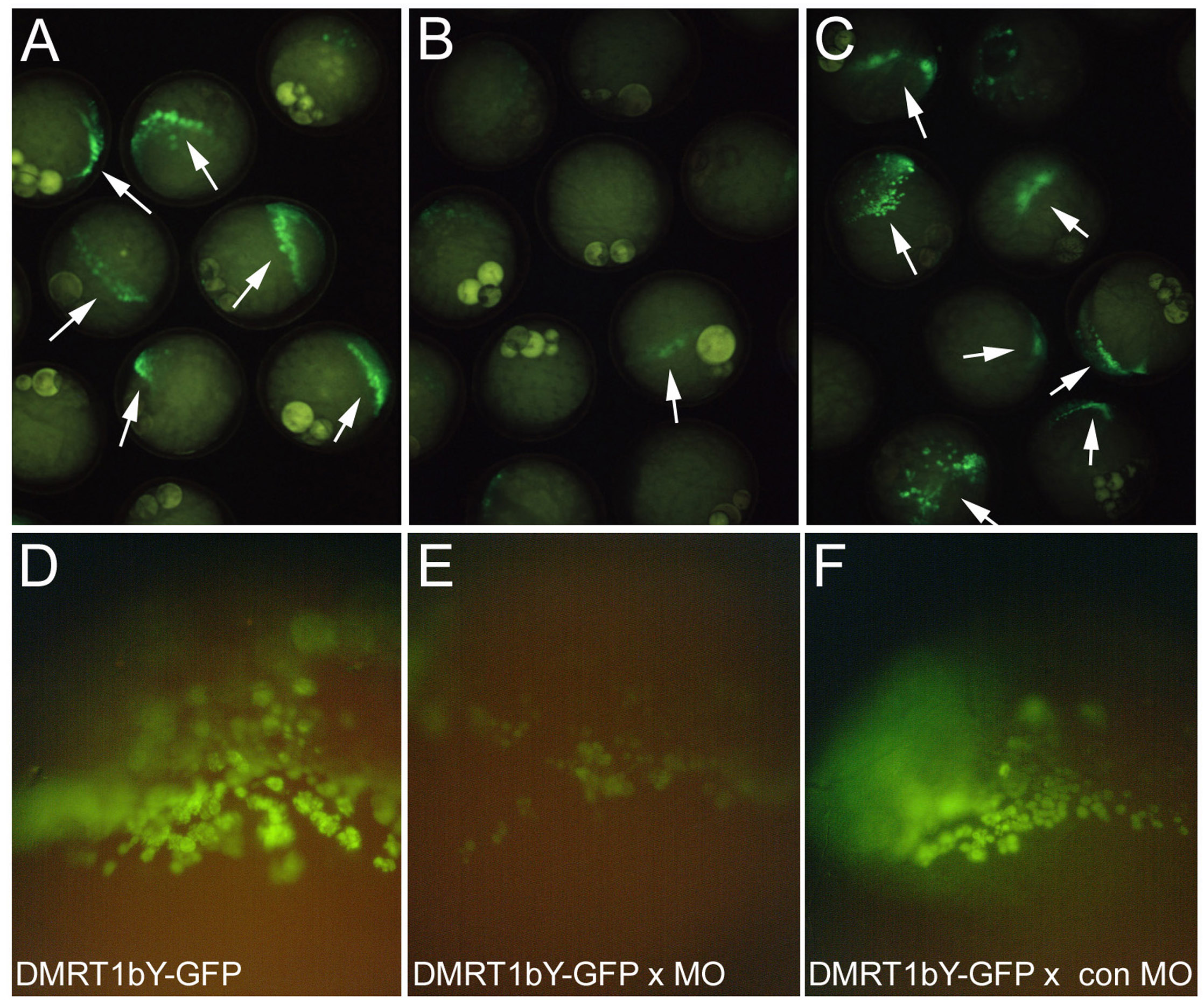

Supplement: Additional file 3 — Analysis of Morpholino efficiency and specificity. 25 pg of a construct containing 557 bp of the upstream DMRT1bY region including UTR and ATG, which drives a GFP reporter (fused in-frame with a DMRT1a cDNA), were injected alone or in combination with 0.85 ng DMRT1bY Morpholino into Medaka embryos at the one to two cell stage. Injection of the DNA alone resulted in strong GFP expression (in 11/12 embryos; arrows in A), which was significantly reduced in embryos coinjected with the DMRT1bY Morpholino (weak GFP expression in 15/22 embryos). GFP expression was not reduced, when a control Morpholino (directed against the autosomal copy of DMRT1) containing five base pair changes compared to the DMRT1bY sequence was coinjected with the DMRT1Y-GFP fusion construct (strong GFP expression in 15/15 embryos; arrows in C). Higher magnification views in (D-F) show nuclear GFP expression, which was predominantly found in the marginal zone of embryos analyzed at 50% epiboly. Comparison of the dmrt1bY specific morpholino sequence to the corresponding sequence in dmrt1a: [file 1471-213X-7-99-S3.jpeg]
